# A comparison of several computational auditory scene analysis (CASA) techniques for monaural speech segregation

**DOI:** 10.1007/s40708-015-0016-0

**Published:** 2015-08-04

**Authors:** Jihen Zeremdini, Mohamed Anouar Ben Messaoud, Aicha Bouzid

**Affiliations:** National School of Engineers of Tunis, LR11ES17 Signal, Image and Information Technology Laboratory, University of Tunis El Manar, 1002 Tunis, Tunisia

**Keywords:** Auditory system, Monaural speech segregation, Computational auditory scene analysis (CASA), Segmentation, Grouping

## Abstract

Humans have the ability to easily separate a composed speech and to form perceptual representations of the constituent sources in an acoustic mixture thanks to their ears. Until recently, researchers attempt to build computer models of high-level functions of the auditory system. The problem of the composed speech segregation is still a very challenging problem for these researchers. In our case, we are interested in approaches that are addressed to the monaural speech segregation. For this purpose, we study in this paper the computational auditory scene analysis (CASA) to segregate speech from monaural mixtures. CASA is the reproduction of the source organization achieved by listeners. It is based on two main stages: segmentation and grouping. In this work, we have presented, and compared several studies that have used CASA for speech separation and recognition.

## Introduction

The auditory system is an acoustic and cognitive wonder. Indeed, it has a remarkable ability to decompose the different sources of soundscape, even noisy, and instantly make sense of this entire noisy environment that reaches our eardrums. In addition, when several speakers are talking simultaneously, we are able to easily follow the speaker of interest. However, this is a problem that remains highly complex in digital signal processing. Indeed, the estimation of superposed signals in a real environment is the current problem posed. For this, several techniques have been developed to achieve the purpose of composite speech separation.

In this context, we mention the blind sources separation (BSS) which is the most general form of source separation problem. It aims to extract the unknown speech signals from the mixture signals without consideration of any “a priori” information on signals sources or on mixture signals. Mixture signals observed at a set of sensors are generally a combination of the source signals which are undergoing changes and were added [1].

Since BSS is only based on multiple sensors records and our interest is on the monaural speech segregation, we will be focusing later only on these approaches. Several methods have been proposed for monaural speech separation, like spectral subtraction [2], subspace analysis [3], hidden Markov modeling [4], and sinusoidal modeling [5]. These approaches usually suppose certain properties of interference and then separate composite speech based on these hypotheses. That is why their capacity for speech segregation is much limited than the human capacity. Thus, we are interested by the study of the computational auditory scene analysis (CASA).

According to Bregman [6], the separation process in the auditory scene analysis (ASA) has two main steps: segmentation and grouping. The first step is to decompose the auditory scene in time–frequency zones or segments which are sound elements having a coherent structure. The second step is to group segments that may result from the same source in auditory streams. The segmentation and grouping mechanisms exploit acoustic features such as harmonicity, coherent envelope, coherent modulation frequency or amplitude…which are based on the intrinsic characteristics of the sound properties. Two types of combination are defined in the ASA: The simultaneous mechanism and the sequential mechanism. The first mechanism allows the assembly of the segments through the frequencies, while the second mechanism incorporates the segments having similar properties in time.

Research in ASA has inspired considerable work to build CASA. CASA is a separation technique aimed to numerically simulate the mechanisms of the human auditory system to separate sources in the same way as do our ears, at least theoretically. Indeed, it is the study of the auditory scene analysis by computational means (reproduction of the ASA in machines). Several researchers have adopted this approach for the separation of sources. This technique involves two main stages: segmentation and grouping [7–10].

The present paper is organized as follows. The second section presents the different CASA stages and the ideal binary mask. The third section describes Major works using CASA for the composite speech separation and recognition. In the fourth section, an evaluation and a comparison are presented for different monaural speech segregation methods. And finally the fifth section concludes this work.

## Computational auditory scene analysis (CASA)

**Fig. 1:**
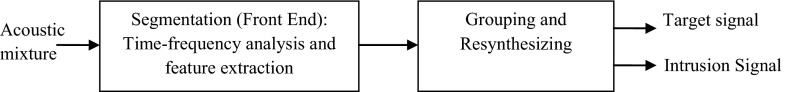
The schematic diagram of the CASA system

Typically, CASA extracts one source from a single channel of audio using heuristic grouping rules based upon psychological observations. Then, it is based on two main stages as ASA: segmentation and grouping [7–10] (Fig. 1).

### Segmentation stage

The first step of CASA system usually consists of a time–frequency analysis of the signal that mimics the frequency selectivity of the human ear and the characteristics extraction which are useful for the following steps. This is the segmentation of the auditory scene in elementary acoustic features [7–10].

Typically, the input signal is passed through a bank of bandpass filters; each one simulates the frequency response associated with a particular position on the basilar membrane. The ‘gammatone’ filter is often used, which is an approximation of the impulse response of the physiologically recorded auditory nerve fibers.

Most CASA systems make the device time–frequency representation and the application of a correlogram to extract features and useful information for the following steps as: the autocorrelation of a filter response, the autocorrelation of a filter response envelope, the cross-channel correlation, the dominant fundamental frequency of each frame…

The filter bank used is generally composed of 128 gammatone filters (or 64 filters) with center frequencies ranging from 80 to 5000 Hz. The impulse response of this filter has the following form:

1 $$g\left( t \right) = \left\{ {\begin{array}{ll} {a{t^{n - 1}}{e^{ - 2\pi bt}}\,{\rm{Cos }}(2\pi f{\mkern 1mu} t + \Phi ),{\mkern 1mu} }&{t > 0}\\ {0,}&{{\rm{else}}} \end{array}} \right.$$

where a is the amplitude, Φ is the phase, *n* is the filter order(we usually take 4), *b* is the filter band width (ER B = 24.7 + 0.108 × *f*), the filter center frequency, t is the time.

For each channel, the output is divided into 20 ms frames with an overlap of 10 ms between two consecutive frames. As a result of this filtering and windowing, the input signal is decomposed into a representation of two-dimensional time–frequency (TF) or a collection of TF units. Now, to extract the acoustic features of the signal, a correlogram which is an autocorrelation executed in each filter response across an auditory filterbank is used. Indeed, it provides an efficient auditory representation mid-level between the auditory periphery and segregation. For each T–F unit, we have:

2 $$A_{H } \left( {c,m,\tau } \right) = \frac{1}{{N_{c} }}\sum\limits_{n = 0}^{{N_{c} - 1}} {h\left( {c,mT - n} \right)h\left( {c,mT - n - \tau } \right)} ,$$

where *c is* the channel, *n is the* step time, *t* is the time delay, *N*_c_: number of samples, *τ* is the delay Є [0, 12.5 ms], *h* (*c*, *n*) is the output of the channel cochlear filter bank.

The correlogram is an effective tool for F0 estimating because it detects the periodicities present in the output of the cochlear filterbank. Indeed, a convenient way to determine F0 consists of adding the correlogram channels as indicates this equation:

3 $$s\left( {m,\tau } \right) = \mathop \sum \limits_{c} A_{H} (c,m,\tau ).$$

The sum of the resulting autocorrelation function has a peak at the period of each F0.

### Grouping stage

After the first stage, we obtain a time–frequency representation in order to extract features that are useful for grouping. The grouping stage presents the problem of determining which components should be grouped together and identified as the same sound. Principal features that are used for grouping are fundamental frequency (F0), harmonicity, onset synchrony, continuity, etc. Then, the signal components are split into groups based on the similarity of their source and location attributes. These groups are the separated signals.

In this context, it can be classified into sequential grouping cues (across time) and simultaneous grouping cues (across frequency) [7–10]:

- Sequential grouping is influenced by many of the factors that define the similarity, the frequency proximity, the repetitive character, and the repetition rate of successive sounds.
- Simultaneous grouping is affected by harmonicity, envelope coherence, binaural correlation, amplitude modulation, and frequency modulation.

### Ideal binary mask (IBM)

The notion of an ideal binary mask (IBM) has been proposed as a primary goal of CASA.

In the time–frequency representation of the front-end part, the key factor behind the notion of ideal binary mask is to keep the time–frequency regions of the target that are stronger than those of the interference, and delete regions which are weaker than the latter. More precisely, the ideal mask is a binary matrix, where “1” indicates that the energy of the target is higher than the energy of the interference inside the corresponding TF unit and “0” indicates the opposite [7–10]:

4 $$M(t,\;f) = \left\{ {\begin{array}{ll} 1 & {{\text{if}}\;s(t,\;f) - n(t,\;f) > \theta}, \\ 0 & {{\text{else}}}. \\ \end{array} } \right.$$

where *s* (*t*, *f*) is the target energy in a TF unit, *n* (*t*, *f*) is the interference energy.

Weintraub was the first who used this approach in a CASA system, which had been adopted by several other researchers. The use of binary masks is motivated by the masking phenomenon of the human ear, in which a weaker signal is masked by a stronger within the same critical band. It is also noted that the reconstruction of a masked signal can be interpreted as a highly nonstationary Wiener filter. The IBM has several properties such as:

- *Flexibility* Depending on the target and with the same mixture, we can define different masks.
- *Good definition* The mask is well defined even if there are several intrusions in the speech mixture and we can also estimate several targets from this same mixture.
- The ideal binary mask is more performant than all existing masks. In fact, it gives excellent resynthesis for a variety of sounds.

### Major works using CASA for the separation of the composite speech

There are several works that have used the CASA system for the composite speech segregation, multiple fundamental frequencies estimation and tracking, speech recognition, etc. All following works are based on CASA system.

For monaural segregation and multi-pitches estimation, we note essentially the approach of Hu and Wang [11] who proposed a system for resolved and unresolved harmonics segregation of voiced speech. For resolved harmonics, the model generates segments based on temporal continuity and cross-channel correlation, and groups them according to common periodicity. In order to segregate unresolved harmonics, authors use the common amplitude modulation (AM) and the temporal continuity to generate segments which will be grouped after according to AM repetition rates.

**Fig. 2:**
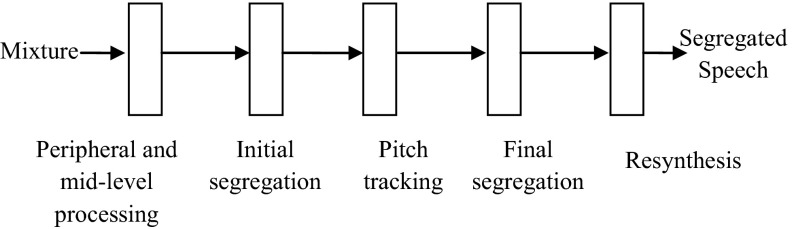
The schematic diagram of the proposed multistage system

The Fig. 2 represents the schematic diagram of the proposed multistage system.

In the first stage, an input signal is decomposed with a bank of 128 gammatone filters into two-dimensional time–frequency units. Then, autocorrelation of a filter response, cross-channel correlation and dominant pitch are extracted for each frame and used in the following stages.

In the second stage, these T–F units are merged into segments which are grouped into initial foreground stream and a background stream based on a dominant pitch. Then, two streams are obtained which respectively correspond to target and intrusion speech.

In the third stage, the fundamental frequency of the target speech is extracted from the initial foreground stream and it is used to mark units as speech dominant or interference dominant.

In the next stage, segments obtained in the initial segregation are regrouped based on unit labels in order to obtain foreground and background streams.

Finally, target speech and intrusion speech are obtained by synthesizing the speech waveform from the resulting foreground stream.

After this work, the same authors [12] proposed a tandem algorithm that performs pitch estimation of a target speech and voiced zones segregation. This algorithm first obtains a rough estimation of target pitch, and then uses this estimation to segregate target speech using harmonicity and temporal continuity. This algorithm improves both pitch estimation and voiced speech segregation iteratively.

On the other hand, Zhang and Liu [13] added to CASA system minimum amplitude and harmonicity principles for resolved harmonics segregation. To segregate unresolved harmonics, they extracted AM rate by the enhanced autocorrelation function of the envelope. The “Enhanced” ACF eliminates the fake period peaks and improves the robustness.

Besides, Zhang and Liu [14] presented a novel approach for monaural voiced speech separation that differs with usual methods by avoiding the compute of correlograms. The typical Front-End processing is applied to the composite speech in order to obtain time–frequency units. After that, the zero crossing rate (ZCR) of the T–F units is used to extract the pitch contour of the target speech. Finally, a comb filter is applied to label each unit as target speech or intrusion.

Furthermore, Radfar and Dansereau [15] introduced a new algorithm called “MPtracker” for pitch frequencies estimation and tracking in order to separate two speakers from their mixture. The pitch frequencies are detected by introducing a novel spectral distortion optimization which takes into account the sinusoidal modeling of the speech signal. The detected pitch frequencies are grouped, separated, and interpolated for obtaining two separated speakers.

In addition, we cite Jiang and Liu [16] who proposed a new monaural speech segregation method by the new implementation of the Gammatone frequency cepstral coefficients (GFCC) which are extracted within each T–F unit and the use of a deep neural networks (DNNs) classifier for the ideal binary mask estimation.

**Fig. 3:**
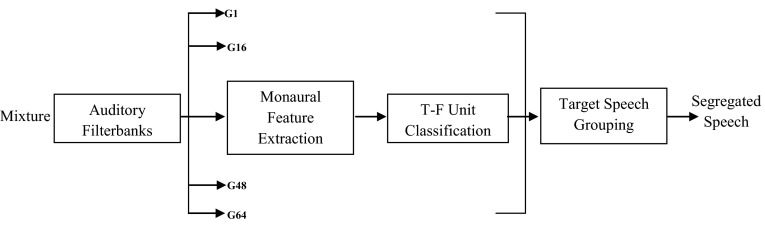
The schematic diagram of the proposed method

Figure 3 shows the diagram of the proposed system.

As CASA system, the input mixture is decomposed into T–F units by the auditory filterbanks. After calculating features for each frame, the GFCC are introduced as the inputs to the binary DNN classifier for each frequency channel. This classifier grouped T–F units to target speech and intrusion speech.

Li and Guan [17] proposed a new method which combines CASA with objective quality assessment of speech (OQAS) in order to segregate voiced speech. In fact, the OQAS algorithm is used to classify foreground and background streams.

This combination introduced the knowledge on speech perceptual quality in separation and constructed a direct link between separated speech and its perceptual quality for improving the performance of the speech separation.

**Fig. 4:**
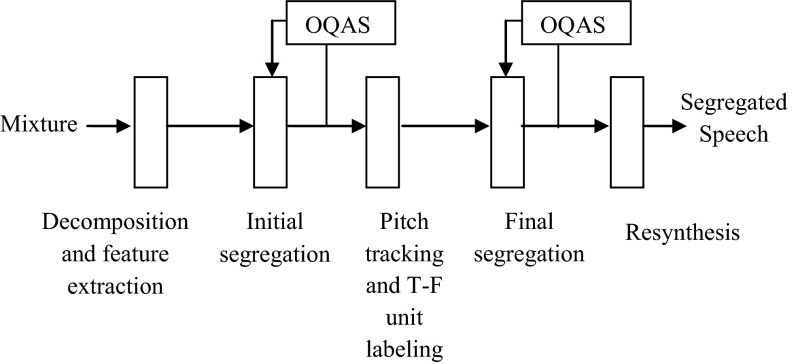
The diagram of the proposed system

The Fig. 4 represents the schematic diagram of the proposed technique.

In this approach, the typical CASA model of Hu and Wang’s system for resolved and unresolved harmonics segregation is employed. For more reliable grouping result of the foreground and background streams corresponding to the target speech and intrusion, there are two parts where OQAS is inserted into this system: in the initial and the final segregation stages.

Hu and Wang [18] used CASA system to segregate unvoiced speech using segregated voiced signals. At first, this system removes estimated voiced speech and the periodic part of interference based on cross-channel correlation. Then, it estimates interference energy by averaging mixture energy in neighboring voiced intervals. Unvoiced speech segregation is decomposed in two stages: segmentation and grouping. In fact, the estimated interference is used by spectral subtraction to extract unvoiced segments, which are then grouped by either simple thresholding or Bayesian classification.

**Fig. 5:**
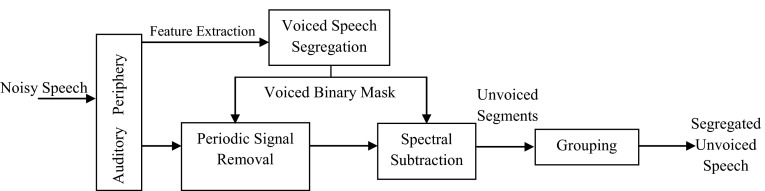
The diagram of the proposed system

Figure 5 shows the diagram of unvoiced speech segregation system.

First, composite speech is analyzed by an auditory periphery model and voiced speech is segregated using the tandem algorithm as Hu and Wang CASA system. After that, the segregated voiced speech is subsequently removed along with the periodic portions of interference from the mixture.

The unit is included in the segregated voiced stream, or it has a high cross-channel correlation.

After the removal of periodic signals, the mixture is composed of only unvoiced speech and a periodic interference. Then, this mixture is segmented by spectral subtraction. Finally, in order to extract only unvoiced speech segments and to remove residual noise, a grouping is carried out.

For speech recognition, Shao and Srinivasan [19] have presented a CASA system for segregating and recognizing the target speech in a mixture. The proposed system is based on two stages. First, the harmonicity is used to segregate the voiced portions of individual sources in each time frame based on multipitch tracking. And an onset/offset analysis is used to segment unvoiced portions. Second, speaker characteristics are used to group the T–F units across time frames. The resulting masks are used in an uncertainty decoding framework for automatic speech recognition.

**Fig. 6:**
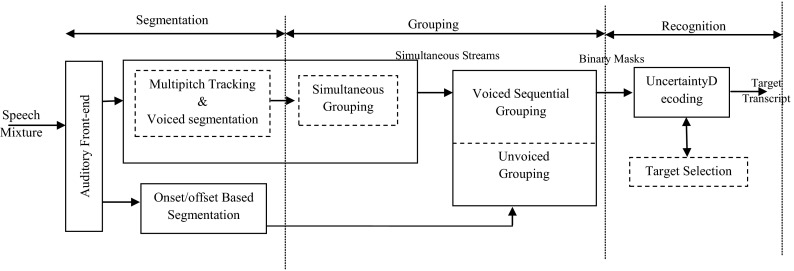
The diagram of the proposed system

The Fig. 6 shows the diagram of proposed system.

The input signal is analyzed by an auditory front-end to obtain T–F representation. In the segmentation stage, both voiced and unvoiced segments are generated. After that, a simultaneous grouping process uses periodicity similarity to group voiced components and produces simultaneous streams. In addition, a sequential grouping algorithm organizes these simultaneous streams and unvoiced segments across time. The resulting binary T–F masks are used by an uncertainty decoder and a target selection mechanism to recognize the target utterance.

Zhao and Shao [20, 21] used CASA as a front-end processor for robust speaker identification (SID).

In fact, they have first introduced the GFCC, based on an auditory periphery model for better speaker characteristics capture. They have also applied CASA masks for speech separation for noisy speech in order to better reconstruct or marginalize corrupted components. Then, they have combined both reconstruction and marginalization methods into their system for best results.

**Fig. 7:**
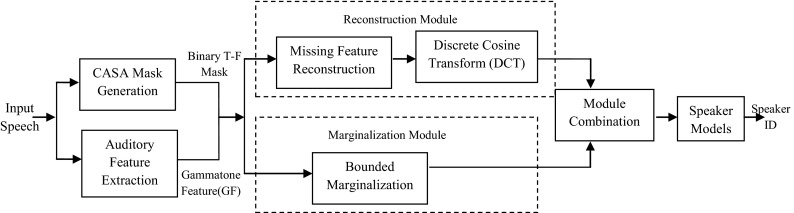
The diagram of the proposed system

Figure 7 shows the diagram of proposed system.

The CASA system is applied to the input signal, in order to compute a binary mask which indicates whether a particular T–F unit is dominated by target speech or by intrusion. In the same time, the input speech is decomposed into gammatone features (GF) by an auditory filterbank. And, GFCC are derived from GF by a cepstral analysis. After that, with CASA masks, unreliable components can be reconstructed or marginalized. As reconstruction and marginalization modules perform well in different conditions, a combination system integrating these two modules is proposed.

## Evaluation and comparison

In this section, we cite only approaches that are evaluated on Cooke database [22]. This database is a collection of composite sounds obtained by mixing ten male voiced speech signals with ten other signals representing a variety of sounds called interferences that can be classified into three categories:

1. Interferences without pitch (N1: White noise and N2: Impulse noise),
2. Interferences having a pitch quality (N0: Pure frequency of 1 kHz, N3: Cocktail party noise, N4: Rock music, N5: Siren and N6: Ringtone).
3. Speech interferences (N7: Speech signal uttered by a woman 1, N8: Speech signal uttered by a man 2 and N9: Speech signal uttered by a woman 2).

### SNR

To evaluate the performance of studied models, the signal-to-noise ratio (SNR) is applied. Its computation is as follows:

5 $${\text{SNR}} = 10 \,{\text{log}}_{10} \left[ {\frac{{\mathop \sum \nolimits_{t} R\left( t \right)^{2} }}{{\mathop \sum \nolimits_{t} \left[ {R\left( t \right) - S\left( t \right)} \right]^{2} }}} \right],$$

where, *R*(*t*) is the clean speech, *S*(*t*) is the synthesized waveform by segregation systems.

The Table 1 contains the SNR results for different methods that are evaluated on Cooke database.

**Table 1:** SNR results

	N0	N1	N2	N3	N4	N5	N6	N7	N8	N9	Average
Mixture	−3.26	−4.07	10.19	4.34	3.99	−5.82	1.90	6.62	10.37	0.73	2.49
Hu and Wang [11]	16.34	7.83	16.71	8.32	10.88	14.41	16.89	11.97	14.44	5.27	12.30
Hu and Wang [12]	24.50	13.50	20.30	13.40	11.99	22.40	18.60	15.11	17.60	8.66	16.60
Zhang and Liu [13]	17.07	5.94	17.26	6.26	8.50	15.18	16.23	11.50	14.43	7.40	11.97
Zhang and Liu [14]	17.86	8.16	18.27	8.26	11.28	16.04	17.46	11.93	14.84	4.98	12.90
Li and Guan [17]	11.13	3.50	14.41	5.21	6.66	12.93	14.66	9.39	11.50	3.96	9.33
True pitch	16.33	8.35	17.71	8.79	11.56	15.06	17.76	12.31	15.32	6.04	12.92
Narrow band [23]	9.88	6.74	11.44	6.94	8.95	8.33	11.31	9.15	10.60	3.98	8.73
Comb filter	3.12	3.01	13.28	8.72	8.32	2.25	6.56	10.57	13.19	5.39	7.44
Wang–Brown [24]	11.31	4.93	11.19	5.65	8.72	10.44	11.15	9.22	10.84	2.66	8.61
Spectral subtraction	18.35	3.05	16.00	6.14	8.32	−5.51	4.85	8.23	10.90	2.46	7.27
Ideal binary mask	20.76	9.04	22.90	9.72	13.19	18.40	21.53	15.78	18.10	10.5	15.99

We compare some of precedent developed methods and other approaches for composite speech segregation. We conclude that the tandem algorithm of Hu and Wang [11] performs consistently better than other systems. In fact, they introduced a new aspect to usual CASA system that treats unresolved harmonics in the high-frequency range. And, they improved pitch estimation and voiced speech segregation using harmonicity and temporal continuity.

This table contains also true pitch that is obtained from premixing target speech and further verified manually to ensure high quality for examining more closely the type of error. Moreover, we cite the Narrow band filter which is an alternative filterbank with a fixed narrow bandwidth and the comb filtering method which extracts a harmonic series using pitch information [23]. Indeed this filter retains target speech and attenuates interference whose frequency components are incompatible with the series target harmonic. The results are not as good as those using an auditory filterbank.

The spectral subtraction method which is a standard method for speech enhancement is also cited. However, because of its well-known deficiency in dealing with no stationary interference, it performs significantly worse than other systems.

Besides, we mention Wang and Brown CASA model that is representative of recent CASA systems [24]. The processing of the Wang–Brown model is similar to the first two stages of Hu and Wang model.

**Fig. 8:**
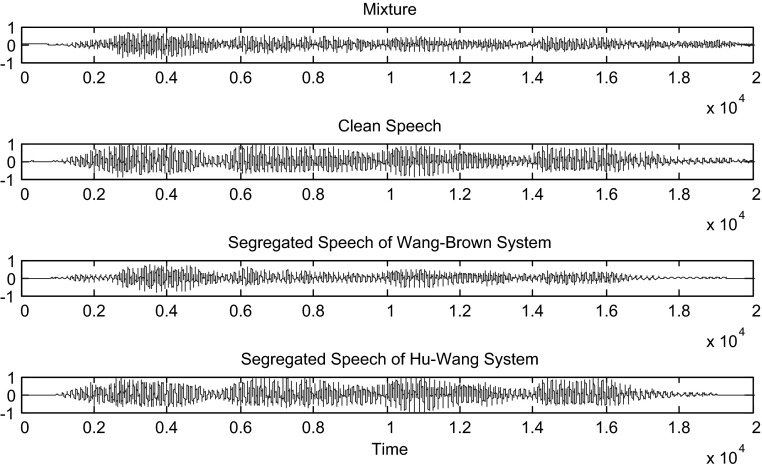
Mixture of the male and female; clean speech, segregated speech from Wang–Brown system and segregated speech from Hu–Wang system

**Fig. 9:**
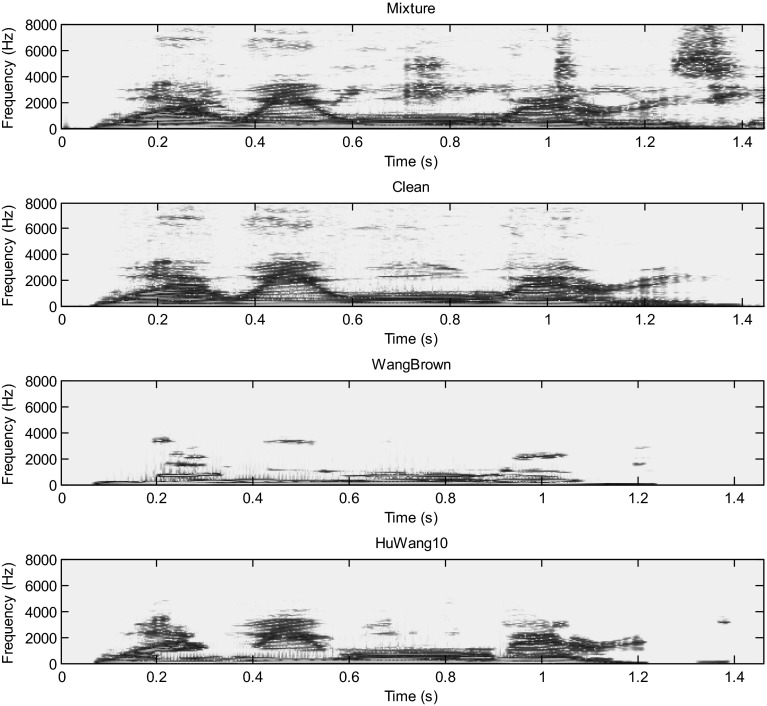
Spectrograms of respectively male and female mixture; clean speech, segregated speech from Wang–Brown system and segregated speech from Hu–Wang system

**Fig. 10:**
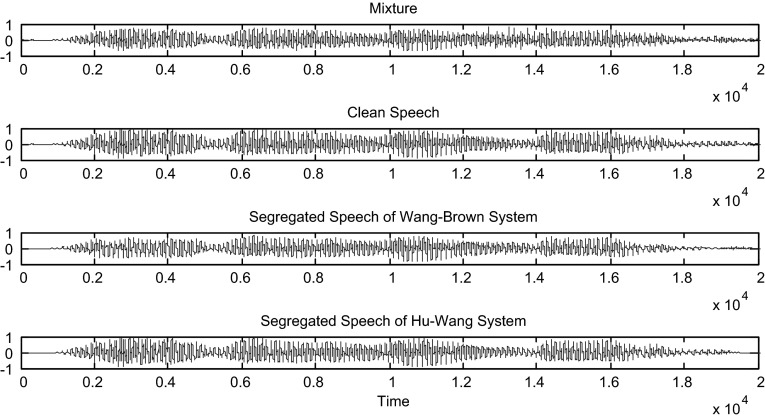
Mixture of two males; clean speech, segregated speech from Wang–Brown system and segregated speech from Hu–Wang system

**Fig. 11:**
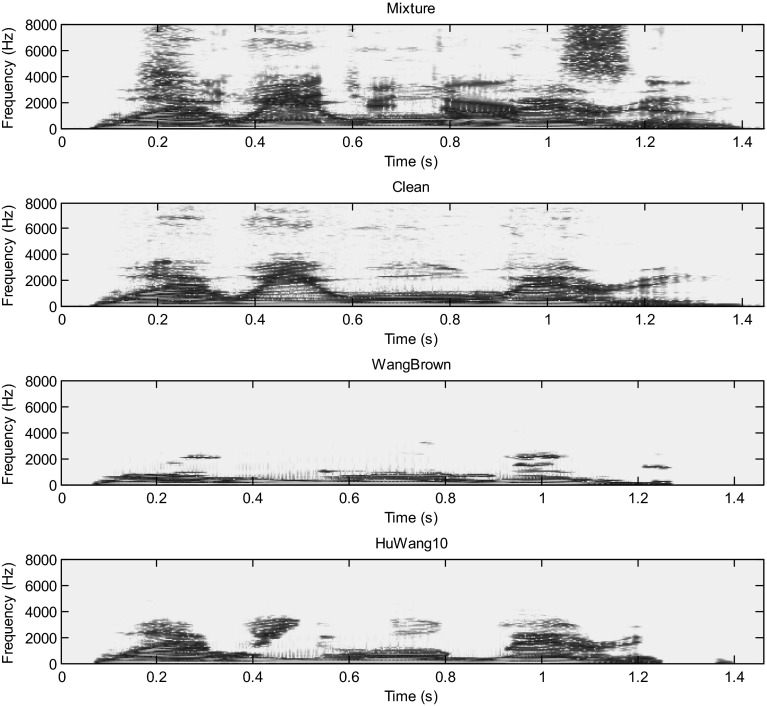
Spectrograms of respectively two males mixture; clean speech, segregated speech from Wang–Brown system and segregated speech from Hu–Wang system

**Fig. 12:**
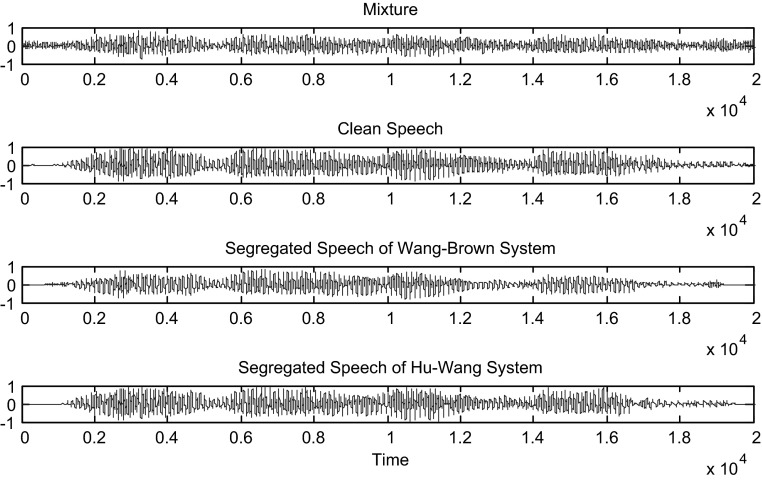
Mixture of the speech and cocktail party noise; clean speech, segregated speech from Wang–Brown system and segregated speech from Hu–Wang system

**Fig. 13:**
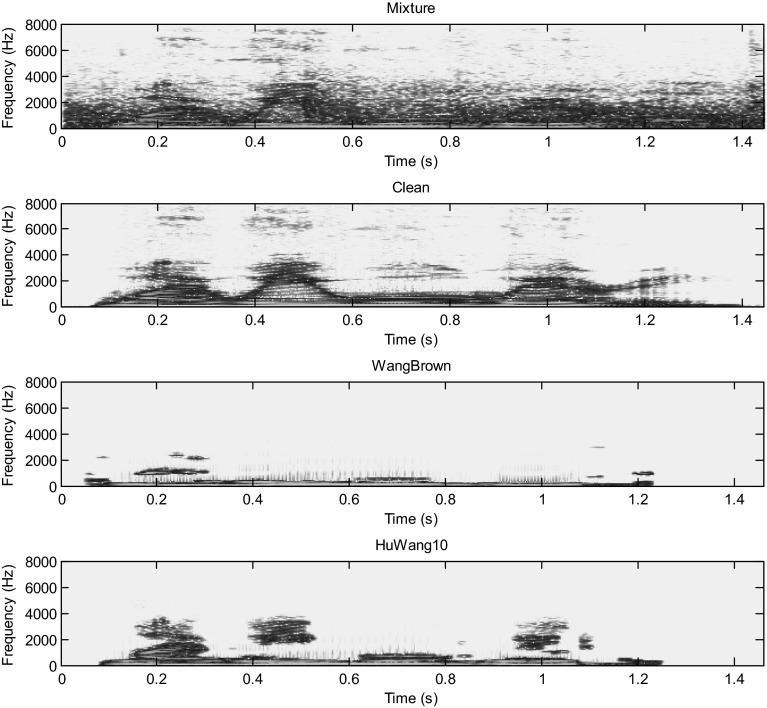
Spectrograms of respectively speech and cocktail party noise mixture; clean speech, segregated speech from Wang–Brown system and segregated speech from Hu–Wang system

Hu and Wang system is more efficient than the Wang–Brown system. In fact, figures below show that the separation is more perfect in the case of Hu. The target signal is more similar to the clean speech (Figs. 8, 9, 10, 11, 12, 13).

From these figures, it is clear that the segregated speech from Hu**–**Wang system is more similar to the clean speech for the three cases than the segregated speech from Wang**–**Brown system.

### Run-time complexity

In this section, we analyze and compare the run-time complexity only of Hu–Wang model to Zhang–Liu system because Hu and Wang model [4] has much better performance than the previous systems.

The entire separation systems are relatively complicated. For this, only the major processes in each stage are compared like correlogram, segmentation, pitch estimation… The complexity of computing correlograms is O (CLlogW), where W is the time frame.

Table 2 shows the different compared processes between Hu and Wang [11] and Zhang–Liu [14].

**Table 2:** Comparison of time complexity

Stage	Process	Hu–Wang [11]	Zhang–Liu [14]
Front-end	Signal decomposition	O(CL)	O(CL)
	Envelope Extraction	O(CLlog(L))	O(CLlog(L))
	Correlograms	O(CLD)	
	ZCR		O(CL)
Pitch estimation	Segmentation	O(CL/T)	O(CL/T)
	Pitch estimation	O(CL)	O(CL)
Unit labeling	Bandpass filtering	O(CLF)	
	Comb filtering		O(CL)
Separation and synthesis		O(CL)	O(CL)

*C* number of channels, *L* length of input signal, *T* time shift, *D* maximum pitch period, *F* length of FIR bandpass filter

In Table 3, we present the computing time of three methods.

**Table 3:** Computing time

	Run time (s)	Real time property
Hu–Wang [11]	2460	14.6 × RT
AccHW	1064	6.33 × RT
Zhang–Liu [14]	375	2.23 × RT

From Table 3, we note that Zhang and Liu system has the best computing time. In fact, the computing time of the first Hu and Wang model [11] is 14.6 times of real time. An enhanced version of Hu and Wang system [11] called “AccHW” consists on calculating the bandpass filtering and correlograms in the spectrum domain. “AccHW” saves 57 % computing time, while the total computing time of the Zhang and Liu model [14] is 2.23 times of real time.

## Discussion and overview

Hu and Wang model [11] has much better performance than previous systems. First, this system applies different mechanisms to deal with resolved and unresolved harmonics. Secondly, the separation is based on segmentation which is more robust than other techniques. Besides, the fundamental frequency is determined in noisy environment and it is applied for final segregation. Moreover, the tandem algorithm of Hu and Wang [12] is robust to interference. In fact, it produces good estimations of both pitch and voiced speech even in the real noisy environment.

Nevertheless, in the case of two-speaker situation (the third category of Cooke database (N7, N8, and N9)), the performance of these methods is relatively limited. In fact, these models make grouping based only on pitch. As a result, they are limited to segregation of only voiced speech. In addition, unvoiced speech presents also a big challenge for monaural speech segregation.

On the other hand, according to Zhang and Liu [13], the Hu–Wang model has failures like AM (amplitude modulation) rate detection error. To overcome the disadvantages, their system uses the “Enhanced” ACF (envelope autocorrelation function) to eliminate the wrong period peaks and to improve the robustness. Added to that, the Zhang and Liu system [14] has the best computing time (see Sect. 4.2).

Besides, for Radfar and Dansereau [15], their algorithm detects and tracks the pitch contours for the dominant and intrusion signals. Besides, this model does not suppose that the mixture signal is only voiced. Also, it assigns the contours of pitch to individual speakers.

In addition, Jiang and Liu method [16] has shown consistent and significant automatic speech recognition (ASR) performance gains in various noise types and SNR level conditions. In fact, this system achieves more robust segregation in low SNR conditions. Nevertheless, the performance decreases gradually in no stationary noisy and reverberant conditions.

Moreover, Li and Guan [17] make a link between CASA system and the speech quality. This combination enables a better selection of the segments which were not affected greatly by interference sources and use them to track the pitch contour which can be useful in the separation step.

However, there are some weaknesses in this approach. First, the model performance depends greatly on the accuracy of an estimated target pitch contour. In fact, the classification of the foreground and the background in the initial segregation stage is mainly based on the objective quality assessment of speech (OQAS) algorithm. But, it is still a machine estimation and the obtained result is more or less different from the subjective mean open score (MOS). In addition, the combination of CASA with OQAS is not the best combination. It is necessary to find an optimal combination method of CASA and OQAS to ameliorate the separation. Moreover, this system just enables voiced speech segregation based only on pitch. It does not address the problem of unvoiced speech separation.

Finally, from the previously presented methods, we conclude that CASA system is introduced to solve the problem of speech segregation by mimicking the auditory process of source separation. In fact, CASA does not make strong assumptions about interference. Also, it can be used on single channel input.

For harmonics segregation, the earlier CASA systems employ the human strategy. These systems have good segregation results for resolved harmonics but poor for unresolved ones. Besides, in high frequency, the performance is not as good as in the low frequency because intrusions are stronger. However, current CASA systems have resolved these problems by applying different mechanisms to deal with resolved and unresolved harmonics and using new techniques which are more robust for the separation process. Nevertheless, the performance of these systems is still limited by fundamental frequency estimation errors, residual noise and in the case of two-speaker situation [10].

## Conclusion

In this paper, we have focused on CASA for monaural speech segregation. CASA is based on two stages: segmentation and grouping. In the segmentation stage, the input mixture is passed through a bank of bandpass filters in order to obtain time–frequency units and the application of a correlogram to extract features that are useful for the following stage. Usually, the ‘gammatone’ filter is used because it is an approximation of the impulse response of the physiologically recorded auditory nerve fiber. In grouping stage, the problem of determining which components should be grouped together and identified as the same sound is resolved. There are several methods that used CASA to separate composite speech such as Hu and Wang, Zhang and Liu, Zhao and Shao, Li and Guan approaches, etc. These methods are developed, evaluated and compared too.

## Prospects

As prospects, we want to propose an approach that ameliorates the monaural speech segregation by ameliorating the method of pitch estimation, dealing well with resolved harmonics and unresolved ones. Besides, we want to do the segregation of a monaural mixture containing more than two speakers.
